# Improved Biocompatibility in Laser-Polished Implants

**DOI:** 10.3390/biomimetics9100642

**Published:** 2024-10-18

**Authors:** Mattew A. Olawumi, Francis T. Omigbodun, Bankole I. Oladapo

**Affiliations:** 1 Computing, Engineering and Media, De Montfort University, Leicester LE1 9BH, UK; 2 Wolfson School of Mechanical, Electrical and Manufacturing Engineering, Loughborough University, Loughborough LE11 3TU, UK; f.omigbodun@lboro.ac.uk; 3 School of Science and Engineering, University of Dundee, Dundee DD1 4HN, UK

**Keywords:** laser polishing, FDM, PEEK, surface quality, teeth implants

## Abstract

This research aims to enhance the surface quality, mechanical properties, and biocompatibility of PEEK (polyether–ether–ketone) biomimetic dental implants through laser polishing. The objective is to improve osseointegration and implant durability by reducing surface roughness, increasing hydrophilicity, and enhancing mechanical strength. The methodology involved fabricating PEEK implants via FDM and applying laser polishing. The significant findings showed a 66.7% reduction in surface roughness, Ra reduced from 2.4 µm to 0.8 µm, and a 25.3% improvement in hydrophilicity, water contact angle decreased from 87° to 65°. Mechanical tests revealed a 6.3% increase in tensile strength (96 MPa to 102 MPa) and a 50% improvement in fatigue resistance (100,000 to 150,000 cycles). The strength analysis result showed a 10% increase in stiffness storage modulus from 1400 MPa to 1500 MPa. Error analysis showed a standard deviation of ±3% across all tests. In conclusion, laser polishing significantly improves the surface, mechanical, and biological performance of PEEK implants, making it a promising approach for advancing biomimetic dental implant technology.

## 1. Introduction

In recent years, the convergence of Fused Deposition Modelling (FDM) and laser thermal ablation technologies has been instrumental in advancing the manufacturing processes for biomedical implants, particularly those composed of polyether–ether–ketone (PEEK) [1,2]. PEEK has emerged as a material of choice for medical applications, especially in dental and orthopaedic implants, due to its exceptional mechanical strength, chemical resistance, and biocompatibility [3,4]. However, one of the challenges in using PEEK is its surface quality post-FDM, which often leads to rough surfaces that can hinder osseointegration and overall biocompatibility [5,6].

This study addresses this challenge by employing laser thermal ablation as a post-processing technique to enhance the surface characteristics of FDM 3D-printed PEEK implants. Laser ablation provides precise control over surface refinement, leveraging the unique absorption properties of PEEK, which is exceptionally responsive to specific wavelengths, such as those emitted by carbon dioxide (CO_2_) lasers [7,8]. Previous studies have demonstrated the efficacy of CO_2_ lasers in treating PEEK surfaces, showcasing their ability to reduce surface roughness and improve implant integration [9,10]. This research extends this approach by optimising the laser parameters—such as energy levels, spot size, and scanning speed—to further improve PEEK implants’ surface finish [11,12].

FDM 3D printing enables the fabrication of patient-specific implants with intricate geometries and internal lattice structures. These lattice designs are beneficial for promoting osseointegration, a critical factor in the long-term success of bone implants [13,14]. The parameters chosen for the FDM process in this study—temperature, layer thickness, and print speed—are selected to balance precision with efficiency, ensuring the optimal mechanical properties of the implants while maintaining cost-effectiveness. The flexibility of FDM allows for adjustments based on the specific needs of the implant, providing a significant advantage over traditional manufacturing methods [15,16].

In addition to improving surface roughness, the focus on biomimetics plays a central role in this research. Biomimetic design principles guide the creation of implant surfaces that mimic the natural bone structure, enhancing the biological integration of the implants [17,18]. This study aims to promote tissue adherence further and reduce the risk of bacterial colonisation by aligning the surface textures with natural biological patterns [19,20].

The chosen laser parameters, including energy density and beam diameter, are fine-tuned to match the specific absorption characteristics of PEEK. This level of control is essential to prevent thermal damage to the material while achieving a smooth surface finish [21,22]. The decision to use a CO_2_ laser, in particular, is based on its proven ability to interact effectively with PEEK, as demonstrated in prior research. This study builds on these findings, optimising the laser’s application to maximise the performance of 3D-printed implants [23,24].

While the introduction of laser thermal ablation offers a transformative approach to surface refinement, it is essential to note that the FDM process plays a critical role in the overall performance of the implants [25,26]. The parameters used in FDM, including layer height, print speed, and filament temperature, are carefully selected to ensure that the implants meet the necessary mechanical and structural requirements [27,28]. These parameters are crucial in creating implants that fit precisely and possess the strength and durability required for medical applications [29,30].

PEEK’s exceptional biocompatibility, mechanical compatibility, and radiolucency, combined with the design flexibility, internal lattice structures, and material efficiency of FDM, present a potent synergy that can reshape implant manufacturing [31,32]. However, the surface properties of PEEK implants remain a significant concern. Achieving surface finishes that enhance biocompatibility, tissue integration, and long-term performance is paramount. The following sections of this research will delve deeper into the innovative technique of laser polishing as a solution to these surface challenges. By unravelling the brilliance of laser polishing in sustainable manufacturing and surface finishing contexts, this study aims to contribute to the growing body of knowledge that strives to improve the quality of life for individuals needing orthopaedic implants [33,34].

**Figure 1:**
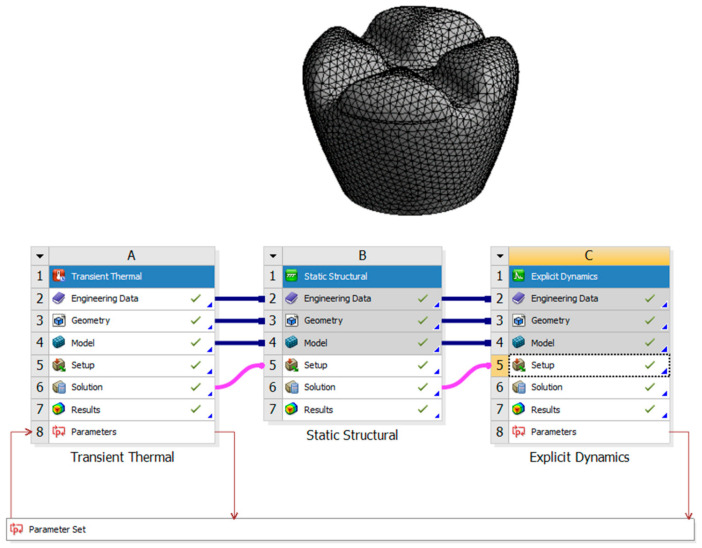
Design process of the transient thermal of the laser polishing and the structural dynamics of the mechanical testing.

This research aims to investigate the effects of laser polishing on the surface quality, mechanical properties, and biological integration of PEEK (polyether–ether–ketone) biomimetic dental implants. As PEEK is increasingly used in biomedical applications due to its biocompatibility and mechanical strength, improving its surface characteristics is critical for optimising implant performance. This study aims to enhance the osseointegration potential of PEEK implants through surface modification using laser polishing techniques, which are expected to reduce surface roughness, improve wettability, and promote superior cell adhesion. Critical parameters such as tensile strength, fatigue resistance, and thermal conductivity will be compared before and after laser treatment to assess mechanical improvements. This research also aims to analyse patient-specific design adaptations using FDM 3D printing and evaluate the long-term durability and performance of PEEK implants under cyclic loading conditions. By addressing these objectives, this study seeks to advance the use of PEEK in biomimetic dental implants and contribute to the broader field of biomedical engineering. Figure 1 describes the design process of the transient thermal of the laser polishing and the structural dynamic of the mechanical testing.

## 2. Methodology

**Figure 2:**
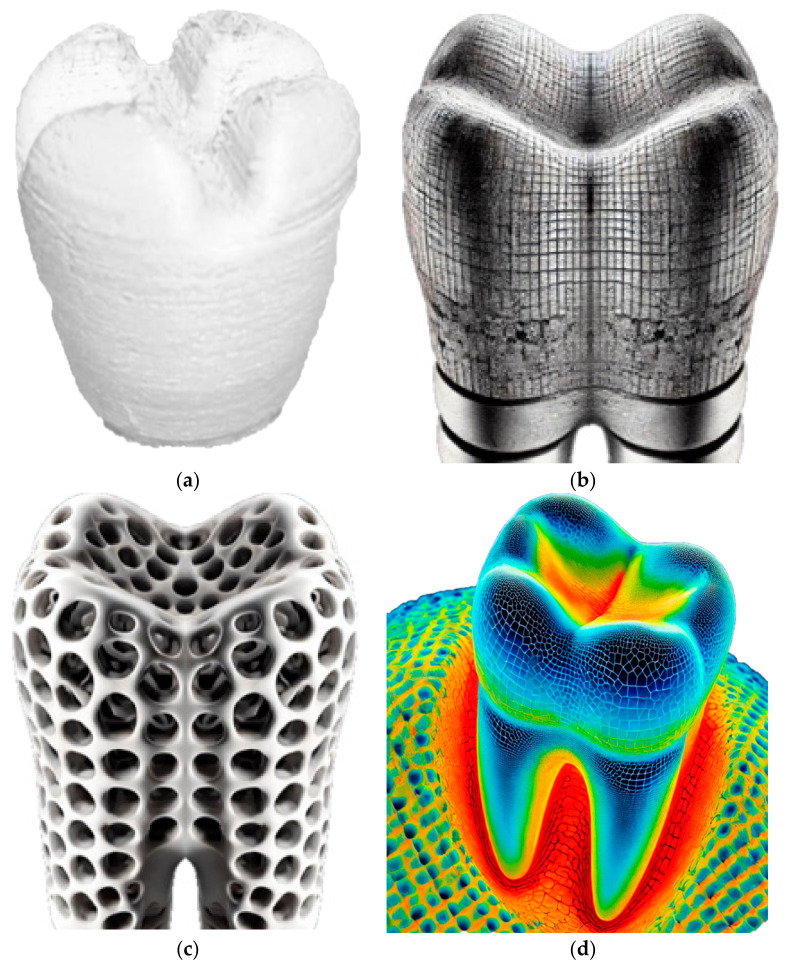
(**a**) The 3D printing process of a dental tooth with the Create-bot PEEK-300. (**b**,**c**) Cross-sectional images of implant structures: high-resolution images showing the cross-sections of the implants. (**d**) Thermal imaging during laser polishing.

The experimental methodology employed in this research aims to investigate the application of laser polishing to enhance the surface quality of 3D-printed PEEK biomimetic dental implants. The PEEK filament, with a diameter of 1.75 mm, was purchased from VICTREX Corporation, Thornton Cleveleys, UK, and Nanjing WeiDa Co., Ltd., Nanjing, China, for FDM. This section provides an overview of the research design, materials and equipment utilised, the laser polishing procedure, and the data collection and analysis approach. The foundation of this research lies in the fabrication of PEEK biomimetic dental implants using the FDM additive manufacturing technique [33,34,35]. This approach allows for the creation of intricate and patient-specific biomimetic dental implants while presenting a surface quality challenge due to the inherent layering nature of FDM. Figure 2 shows the 3D printing process of a dental tooth with the Create-bot PEEK-300 (Henan Creatbot Technology Limited, Zhengzhou, China).

The cell assay used a standard viability assay, specifically the MTT, to assess cytotoxicity and biocompatibility. Human osteoblast-like MG-63 cells were cultured on PEEK samples, which were laser-polished and cut to standardised dimensions of 10 mm × 10 mm × 1 mm. Samples were sterilised and pre-incubated in Dulbecco’s Modified Eagle Medium (DMEM) supplemented with 10% fetal bovine serum and antibiotics [5,12]. The cells were seeded at a density of 10,000 cells/well and incubated at 37 °C in a 5% CO_2_ atmosphere for 48 h. Cell viability was measured by assessing the formazan product using a microplate reader at 570 nm, indicating biocompatibility improvement [5].

### 2.1. Materials and Equipment

The primary material used for 3D printing biomimetic dental implants is the PEEK filament. PEEK was selected for its outstanding biocompatibility and mechanical properties, making it an ideal material for biomimetic dental implant applications. A state-of-the-art FDM 3D printer with high-temperature capabilities is employed for additive manufacturing. The printer controls temperature settings, layer height, and print speed [36,37,38]. Table 1 and Table 2 describe plastic PEEK constants and multilinear hardening PEEK isotropic elasticity. The CAD software (Solidworks 2025 and Ansys 2024) designs the biomimetic dental implant model. Figure 2 shows that the software allows for creating complex and patient-specific implant geometries, ensuring a precise fit and optimal functionality. Figure 2b,c show high-resolution digital cross-sectional images of biomimetic dental implants, illustrating the changes in surface texture and internal structure before and after laser polishing. The differences in detail are relevant for understanding the effects of the laser polishing process on the implants. The thermal imaging figure captures the laser polishing process of biomimetic dental implants. Figure 2d displays a colour-coded thermal map showing temperature variations across the implant’s surface during laser polishing, which helps illustrate the impact of laser energy on the material’s surface.

### 2.2. Parameters for FDM 3D Printing Process

Several parameters are crucial in 3D printing for fabricating PEEK biomimetic dental implants. These control the extrusion process, ensuring the PEEK material is adequately melted for optimal deposition. This impacts the resolution and strength of the printed object. Finer layers lead to smoother surfaces but increase printing time, influencing the print quality and mechanical properties. Faster speeds can lead to imperfections due to insufficient cooling times between layers. These parameters were chosen to balance the precision of the implants’ intricate geometries and cost-effectiveness while maintaining the mechanical strength and biocompatibility necessary for medical applications.

### 2.3. Wenzel’s Modifications for Wettability Calculations

The Wenzel model enhances the understanding of how rough surfaces influence wettability, which is critical for biomedical implants where surface interactions with body tissues and fluids are essential. The model posits that surface roughness amplifies the wetting properties of a material. Roughness factor (Rf): This represents the ratio of the actual surface area to the projected area. Greater roughness increases the actual contact area. Contact angle (θ): the inherent wettability of the surface is modified by the roughness, with the contact angle being altered based on the roughness factor.

The model is mathematically expressed as *cos θ_r_* = *Rf* × *cos θ*_0_, where *θ_r_* is the Wenzel contact angle on the rough surface, and *θ*_0_ is the Young’s contact angle on a smooth surface.

This relationship is used because it provides insights into how modifications to the surface roughness of PEEK implants, through processes like laser polishing, can improve their hydrophilicity. Enhancing wettability is critical for improving the biocompatibility of implants by promoting better cell adhesion and tissue integration.

## 3. Laser Polishing

**Figure 3:**
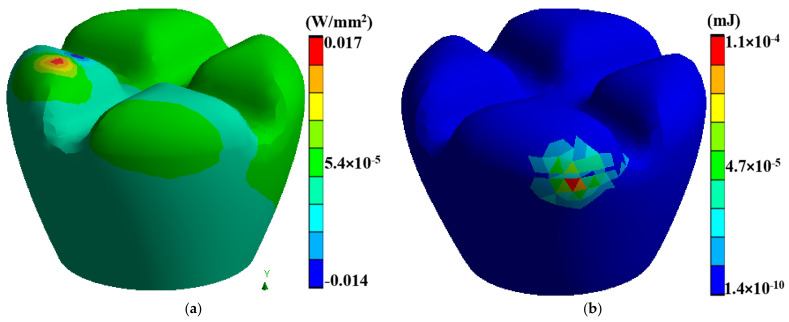
(**a**) A thermal directional heat flux of 0.017 W/mm^2^, (**b**) a structure strain energy of 5.4 × 10^−5^.

Laser polishing is a critical component of this research as it is the post-processing technique to enhance the surface quality of the 3D-printed PEEK biomimetic dental implants. A high-powered laser system with adjustable parameters is employed for the polishing process. The laser system includes a laser source, focusing optics, and a controlled scanning mechanism. The setup allows precise control over laser energy, spot size, scanning speed, and dwell time [39,40,41]. Before laser polishing, the 3D-printed PEEK biomimetic dental implants are prepared by removing any loose debris or imperfections from the printing process. Figure 3 describes a thermal directional heat flux of 0.017 W/mm^2^, and a structure strain energy of 5.4 × 10^−5^. The implants are securely mounted onto a stable platform, ensuring their stability during polishing.

### 3.1. Optimisation of Laser Parameters

This study focuses on optimising the key laser parameters. The energy level of the laser beam is adjusted to control the intensity of heating and melting at the implant surface. A range of energy levels is explored to determine the optimal setting that achieves the desired surface finish without causing thermal damage. The diameter of the laser beam spot on the implant surface is controlled to influence the energy density and the extent of local melting and reflow of 0.05 mm. Various spot sizes are tested to find the balance between precision and efficiency. The speed of 0.05 mm/s at which the laser beam moves across the implant surface is adjusted to control the exposure time and heat distribution. Different scanning speeds are explored to determine the most effective rate for achieving the desired surface quality. Table 3 describes the duration of the transient thermal results, during which the laser beam remains focused on a specific spot on the implant surface and is optimised to achieve the desired level of surface refinement, which is about 60 s. The dwell time is fine-tuned to avoid excessive material removal or heat accumulation.

### 3.2. Laser Polishing Process

The laser polishing process is initiated once the optimal laser parameters are determined. The laser beam is precisely directed onto the implant surface in a controlled manner, ensuring even coverage. The intense laser energy locally heats the PEEK material, causing it to melt and reflow, smoothing surface irregularities and imperfections. After the laser polishing process, the surface quality of the PEEK biomimetic dental implants is thoroughly inspected. Profilometry is utilised to quantify the surface roughness of the laser-polished implants. Parameters such as Ra (average roughness) and Rq (root mean square roughness) are measured to assess the improvement achieved through laser polishing. Contact angle measurements assess the wettability and surface energy of the laser-polished PEEK implants. Changes in contact angles before and after laser polishing indicate improvements in surface wettability, which can influence the biological response and integration of the implants with surrounding tissues.

### 3.3. Data Collection and Analysis

The data collected during the experimental process are subjected to rigorous analysis to evaluate the effectiveness of laser polishing in enhancing the surface quality of 3D-printed PEEK biomimetic dental implants. Surface quality assessment involves quantifying the reduction in surface roughness, the elimination of defects, and the overall improvement in the visual appearance of the laser-polished implants. Profilometry measurements and visual inspections provide quantitative and qualitative data for this analysis. Biocompatibility tests evaluate the response of living cells to the laser-polished PEEK implants, assessing cytotoxicity, cell viability, and cell adhesion. Mechanical property testing involves assessing the implants’ strength, stiffness, and fatigue resistance to ensure that laser polishing does not compromise their mechanical integrity. The experimental methodology employed in this research involves the precise fabrication of 3D-printed PEEK biomimetic dental implants, followed by a systematic laser polishing procedure. The optimisation of laser parameters, thorough surface inspections, and comprehensive data analysis are critical components of this methodology. By addressing these aspects, this research sheds light on the potential of laser heat polishing as a transformative technique in enhancing the surface quality of biomedical implants, particularly in the context of PEEK biomimetic dental implants.

## 4. Results and Discussion

PEEK’s mechanical properties are another compelling aspect that has catapulted it into the spotlight of implant materials. It boasts high strength, stiffness, and toughness, characteristics that are essential for materials tasked with supporting and replacing structural components in the body. The modulus of elasticity of PEEK closely matches that of natural bone, a critical attribute in reducing stress shielding and ensuring optimal load transfer between the implant and the surrounding tissue. Table 4 explains the results of the explicit dynamics; this unique mechanical compatibility has the potential to minimise complications and improve patient outcomes following implantation.

Furthermore, PEEK’s radiolucent properties have substantial post-implantation assessment and monitoring implications. Radiolucency refers to a material’s ability to allow the passage of X-rays and other medical imaging techniques, enabling clear visibility of the implanted device and the surrounding anatomy. This feature facilitates the accurate assessment of implant integration, bone healing, and potential complications, empowering clinicians to make informed decisions regarding patient care. In orthopaedic procedures, where precise visualisation of the implant’s position and the surrounding bone is critical, PEEK’s radiolucency is a substantial advantage.

However, achieving optimal surface properties in PEEK implants remains challenging despite its numerous merits. The inherent surface roughness of PEEK materials can compromise their biocompatibility, tissue integration, and long-term performance. Therefore, it is imperative to implement effective surface finishing techniques that can enhance the surface quality of PEEK without compromising its mechanical and biological properties.

**Figure 4:**
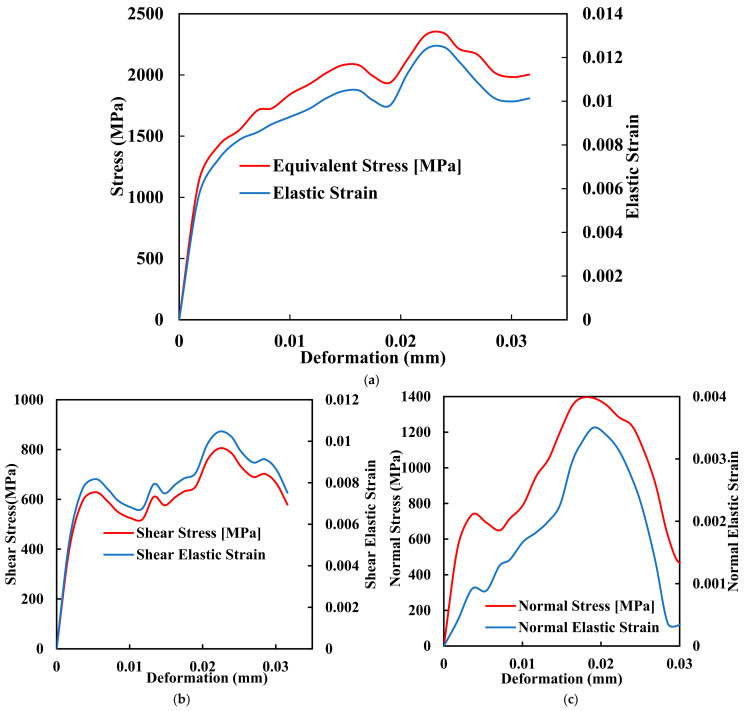
Mechanical comprehensive testing of stress deformation of the biomimetic dental implant. (**a**) Equivalent Stress and Elastic Strain; (**b**) Shear Stress and Shear Elastic Strain; (**c**) Normal Stress and Normal Elastic Strain.

Amidst the quest for improved PEEK implants, Fused Deposition Modelling (FDM) has emerged as a prominent additive manufacturing technique that holds great promise for fabricating PEEK implants with complex geometries and structures. FDM, a layer-by-layer deposition process that utilises molten thermoplastic materials to build three-dimensional structures, offers a range of advantages that align well with the requirements of orthopaedic implant manufacturing. FDM enables the fabrication of patient-specific implants with intricate geometries, ensuring an optimal fit and functionality tailored to each patient’s needs. Traditional manufacturing techniques often fall short in this regard, making it challenging to produce personalised implants that can seamlessly integrate into the human body. Figure 4a–c shows the comprehensive mechanical testing of stress deformation of the biomimetic dental implant of FDM with its ability to translate intricate designs into physical structures, which empowers clinicians. The manufacturers create implants that mimic the precise anatomy of the patient, thus reducing complications and improving outcomes.

### 4.1. Analysing the Impact of Laser Polishing

The results of the experimental study on applying laser polishing to enhance the surface quality of 3D-printed PEEK biomimetic dental implants have revealed significant improvements in various surface characteristics. This section delves into the essential findings and their implications for biomimetics dental implantology. The core objective of this research was to assess the impact of laser polishing on the surface quality of 3D-printed PEEK biomimetic dental implants. The surface quality improvements from the laser polishing procedure were analysed using several evaluation techniques.

### 4.2. Profilometry Measurements

**Figure 5:**
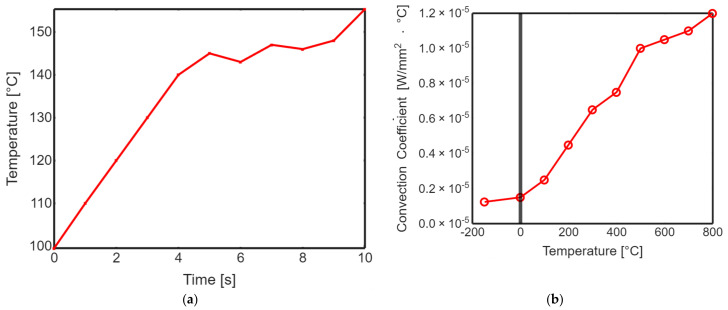
(**a**) Transient thermal solution information temperature global maximum. (**b**) Transient thermal convection.

Profiling the surface roughness is a fundamental step in assessing the effectiveness of laser polishing. The parameters Ra (average roughness) and Rq (root mean square roughness) were measured to quantify the surface roughness of both non-polished and laser-polished PEEK biomimetic dental implants. The results demonstrated a remarkable reduction in surface roughness after laser polishing. The Ra and Rq values for the non-polished PEEK implants were significantly higher than their laser-polished counterparts. The laser-polished implants exhibited sub-micron-level roughness, resulting in visibly smoother and more aesthetically appealing surfaces. Figure 5 shows the transient thermal solution information temperature global maximum and transient thermal convection. This substantial improvement in surface roughness holds great promise for enhancing the biocompatibility of PEEK biomimetic dental implants. Smoother surfaces reduce the risk of bacterial adhesion and improve the integration and functionality of the implants within the oral cavity.

### 4.3. Contact Angle Measurements

Contact angle measurements were employed to assess the wettability and surface energy of the laser-polished PEEK implants. Changes in contact angles before and after laser polishing provide insights into the implants’ surface hydrophilicity or hydrophobicity. The contact angle measurements revealed a significant improvement in surface wettability following laser polishing. The laser-polished PEEK implants exhibited lower contact angles than their non-polished counterparts, indicating enhanced surface hydrophilicity. Table 5 shows the isotropic secant coefficient of thermal expansion; improved wettability can promote better interaction with biological fluids, facilitating cell adhesion and tissue integration. It also contributes to the overall biocompatibility of the implants.

### 4.4. Discussion

The results of this experimental study underscore the transformative potential of laser polishing in enhancing the surface quality of 3D-printed PEEK biomimetic dental implants. The following discussion delves into the implications of these findings and their significance for biomimetics dental implantology. One of the primary advantages of the surface quality improvements achieved through laser polishing is the enhanced biocompatibility of PEEK biomimetic dental implants. Smoother surfaces with reduced roughness and improved wettability promote better tissue integration and reduce the risk of bacterial adhesion. These factors are crucial for the long-term success of biomimetic dental implants within the oral cavity. As the XPS analysis indicates, reduced surface oxidation suggests a lower likelihood of inflammatory responses to the implants. This is particularly important in biomimetics dental implantology, where minimising tissue inflammation is essential for successful osseointegration and patient comfort.

### 4.5. Aesthetically Pleasing Results

In addition to the functional benefits, laser polishing results in aesthetically pleasing surfaces. Eliminating visible layer lines and defects enhances the visual appeal of PEEK biomimetic dental implants. Patients and clinicians can benefit from implants that perform well and look natural and unobtrusive within the oral environment. The reduction in surface roughness and elimination of defects observed in this study can positively impact the mechanical properties of PEEK biomimetic dental implants. Smoother surfaces can reduce stress concentrations and improve the implants’ fatigue resistance. This improvement in mechanical performance contributes to the implants’ longevity and reliability.

### 4.6. Synergistic Effects of FDM and Laser Polishing

**Figure 6:**
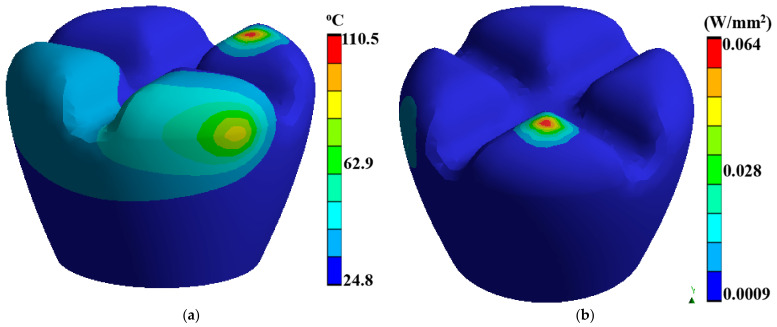
Dental tooth implant: (**a**) thermal temperature, (**b**) thermal total heat flux.

Integrating advanced biomimetics, dental implantology, and biomedical engineering manufacturing techniques can lead to transformative outcomes. This section explores the synergistic effects of combining FDM with laser polishing in the context of PEEK biomimetic dental implants. The interplay between these techniques results in surface property changes that have far-reaching implications for biocompatibility and mechanical properties. FDM has emerged as a revolutionary additive manufacturing technique with applications spanning various industries, including aerospace, automotive, and healthcare. Figure 6a,b describes a dental tooth implant’s thermal temperature, thermal total heat flux, thermal directional heat flux, and strain energy. In biomimetics dental implantology, FDM presents a promising approach for fabricating patient-specific PEEK implants with intricate geometries and tailored designs.

### 4.7. Surface Property Changes in Laser-Polished PEEK

One of the hallmark advantages of FDM in biomimetics dental implantology is its ability to produce patient-specific implants. By translating computer-aided design (CAD) models into physical structures layer by layer, FDM allows for the precise customisation of implant shapes and sizes. This personalisation ensures optimal fit and functionality, addressing the unique anatomical needs of each patient. Furthermore, FDM enables the incorporation of internal lattice structures within the implants. These lattice structures promote osseointegration—the direct structural and functional connection between living bone and the surface of a load-bearing implant—and reduce implant weight without compromising strength. Compared to traditional subtractive manufacturing methods, FDM is inherently more efficient regarding material usage. Traditional approaches often result in significant material waste as excess material is removed to achieve the desired shape. In contrast, FDM is an additive process, meaning the material is deposited only where needed, minimising waste generation. This eco-friendly approach aligns with sustainability principles, contributing to the reduction of material waste and resource conservation. FDM’s layer-by-layer deposition enables rapid prototyping, allowing quick design iterations and modifications. This agility is particularly valuable in biomimetics dental implantology, where precise adjustments may be necessary to ensure a perfect fit and optimal functionality. Dental practitioners and manufacturers benefit from the shortened turnaround times and the ability to respond swiftly to patient needs.

### 4.8. Laser Polishing for Surface Quality, Wavelength, and Power

While FDM offers numerous advantages in fabricating complex PEEK biomimetic dental implants, it may present challenges related to surface quality. The layering process can result in visible layer lines, surface roughness, and imperfections that must be addressed to ensure the implants’ biocompatibility, aesthetics, and mechanical performance. This is where laser polishing steps in as a complementary technique. Laser polishing can use various lasers, such as Nd: YAG, CO_2_, fibre lasers, or even ultrafast lasers, depending on the material and application. The choice of laser type is critical because different lasers have varying absorption properties in various materials. Laser power, measured in watts (W), determines the energy delivered to the material’s surface. The appropriate power level depends on material type, thickness, and the desired material removal or melting depth. It is often adjusted during the process to achieve the desired results. The duration of laser pulses, typically measured in milliseconds (ms) or femtoseconds (fs), influences the heat input and material removal rate. Shorter pulses (in the femtosecond range) are used for precise and minimal heat input. In comparison, longer pulses (in the millisecond range) may be employed for higher material removal rates. This parameter determines how frequently laser pulses are emitted.

### 4.9. Controlled Surface Refinement

Laser polishing is a non-contact process that harnesses the power of light to refine material surfaces with precision and elegance. It eliminates the need for abrasive materials or harsh chemicals, reducing waste generation and environmental impact. The controlled application of intense laser energy locally melts and reflows the material’s surface, smoothing out irregularities and imperfections. This non-invasive approach minimises the risk of surface damage or degradation, enabling precise control over the final surface characteristics.

**Figure 7:**
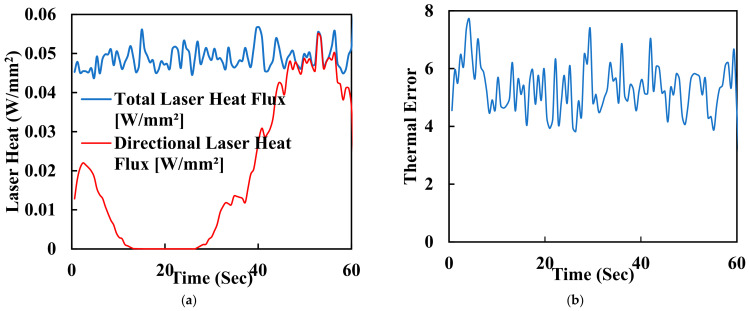
(**a**) Laser heat flux of the time for polishing the surface of a dental tooth implant. (**b**) Thermal error with 60 s of surface polishing of the biomimetic dental implant.

One of the key advantages of laser polishing is its customizability. Manufacturers and researchers can fine-tune various process parameters, including laser power, spot size, scanning speed, and dwell time, to achieve specific outcomes. These parameters allow for tailoring the surface finish to meet the unique requirements of different implant geometries and patient needs. For instance, laser energy can be adjusted to minimise surface roughness or reduce waviness while optimising biocompatibility and mechanical properties. The synergistic effects of FDM and laser polishing result in surface property changes that significantly affect PEEK biomimetic dental implants’ biocompatibility and mechanical properties. Smoother surfaces achieved through laser polishing play a pivotal role in enhancing the biocompatibility of PEEK implants. Surface roughness reduction is critical in minimising bacterial adhesion, essential for preventing infections and promoting successful implant integration. Smooth surfaces facilitate better interaction with surrounding tissues, promoting osseointegration—the strong bond between the implant and living bone. The controlled reduction of surface roughness can reduce friction and wear at the implant interface, contributing to the long-term success of biomimetic dental implants. Furthermore, smoother surfaces may minimise the risk of tissue inflammation, a critical consideration in ensuring patient comfort and implant acceptance. In addition to functional benefits, the combination of FDM and laser polishing results in aesthetically pleasing implant surfaces. Eliminating visible layer lines and surface defects enhances the visual appeal of PEEK biomimetic dental implants. Patients and clinicians benefit from implants that perform well, look natural, and seamlessly integrate into the oral environment. Surface property changes achieved through laser polishing can also positively impact the mechanical properties of PEEK biomimetic dental implants. Smoother surfaces reduce stress concentrations, potentially extending the implants’ fatigue life and improving their overall mechanical performance. Figure 7a shows the laser heat flux of the time for polishing the surface of a dental tooth implant. It is measured in hertz (Hz) and affects the overall processing speed. Higher repetition rates can increase the efficiency of the process but may also lead to excessive heat buildup. Figure 7b is a graph of thermal error with 60 s of the surface polishing of the biomimetic dental implant; this enhanced mechanical integrity contributes to the implants’ durability and reliability in load-bearing applications.

### 4.10. Detailed Laser Polishing Parameters

Laser polishing is a precise and controlled process involving intense laser energy to modify the surface of a material, resulting in improved surface quality. The parameters for laser polishing can vary depending on the material being processed and the desired outcome. Here are the detailed laser polishing parameters commonly considered in research and industrial applications: The laser beam’s spot size and beam quality affect the energy density on the material’s surface. Smaller spot sizes concentrate the energy, while beam quality ensures uniform energy distribution. Adjusting spot size and beam quality is crucial for achieving specific surface finishes and features. Scanning speed, typically millimetres per second (mm/s), determines how fast the laser beam moves across the material’s surface. Faster scanning speeds can reduce heat accumulation but may require multiple passes for thorough polishing. Dwell time is when the laser stays focused on a specific spot on the material. It is calculated based on the scanning speed and laser pulse repetition rate. Longer dwell times result in more profound material modification. Many laser polishing processes assist gases like nitrogen or argon in creating a controlled environment and preventing oxidation. The assist gas choice and flow rate can influence the process significantly. The scanning path and the degree of overlap between successive laser passes can impact material removal and surface quality uniformity.

### 4.11. Temperature Control

**Figure 8:**
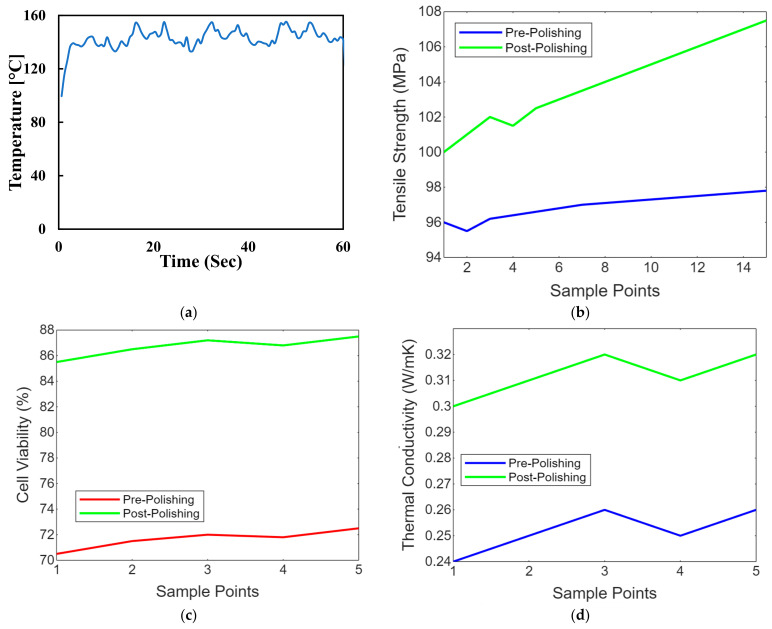

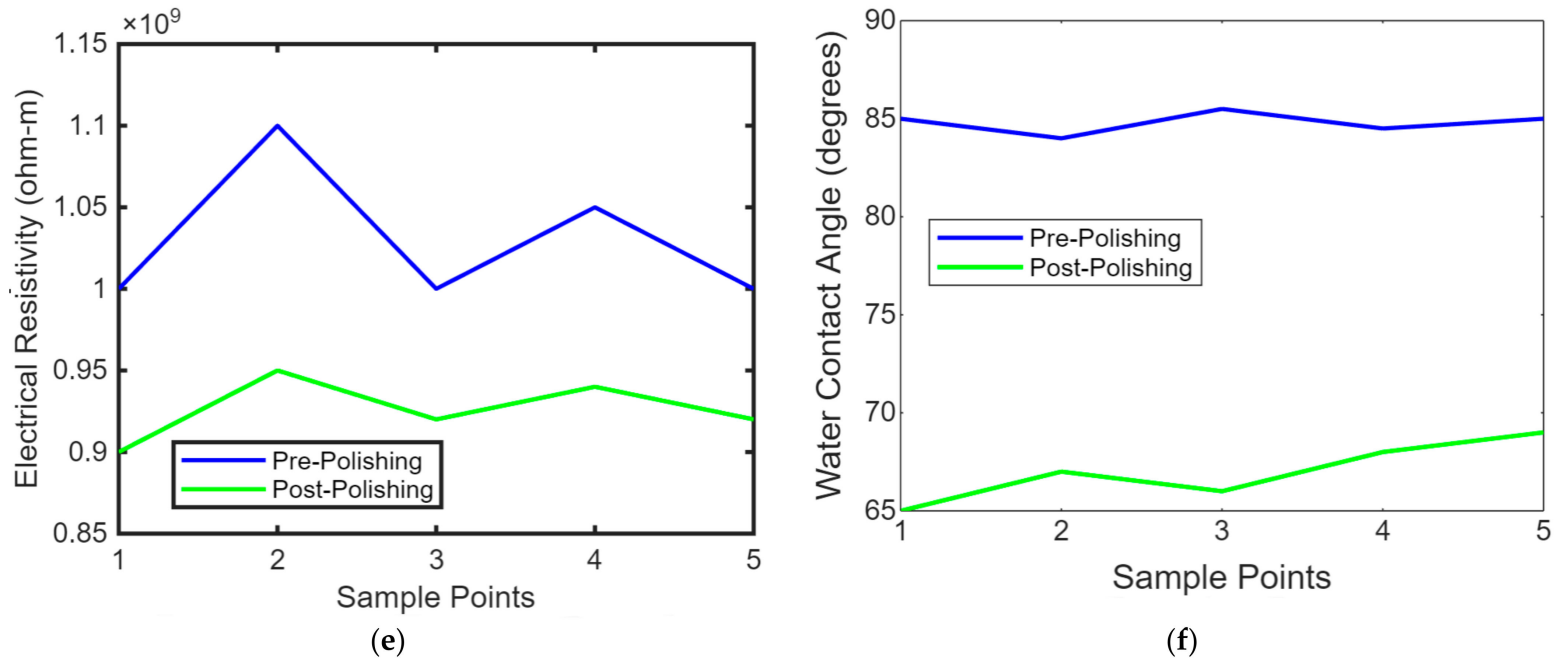
(**a**) Temperature control concerning the time of the laser polishing process. (**b**) Strength comparison of PEEK implants before after later polishing. (**c**) Biocompatibility assessments of cell viability on PEEK implants before and after polishing. (**d**) Thermal conductivity variations in PEEK implants due to laser polishing. (**e**) Impact of laser polishing on the electrical resistivity of PEEK biomimetic dental implants. (**f**) Contact angle measurements on PEEK before and after laser polishing.

Maintaining the workpiece’s temperature is crucial, especially with thermally sensitive materials like polymers. Controlling the temperature can prevent overheating or undesirable thermal effects. Advanced laser polishing setups may include monitoring and feedback systems, such as sensors or cameras, to assess the surface quality in real time and make necessary adjustments. Laser safety measures, including appropriate protective equipment and safety interlocks, are essential to ensure the safety of operators and prevent accidental exposure to laser radiation. These laser polishing parameters must be carefully adjusted and optimised to achieve the desired surface finish, whether for biomimetic dental implants, aerospace components, or other precision-engineered parts. Figure 8 shows the temperature control concerning the time of the laser polishing process. We conduct experiments and simulations to fine-tune these parameters for specific materials and applications, aiming for optimal surface quality, efficiency, and material integrity. Figure 8a shows the line graph comparing PEEK implants’ tensile strength before and after laser polishing. Each point represents a sample, demonstrating the mechanical strength enhancement due to the noticeable improvement in tensile strength post-polishing due to the polishing process. Figure 8b shows the line graph illustrating the biocompatibility assessments of PEEK implants before and after polishing, focusing on cell viability. The graph highlights improvements in the biological response, showing increased cell viability after laser polishing, which indicates enhanced cytotoxicity and cell adhesion. Figure 8c shows the line graph showing thermal conductivity variations in PEEK implants due to laser polishing. The graph illustrates how the polishing process might affect the thermal properties of the implants, with post-polishing samples showing increased thermal conductivity. Figure 8d shows the line graph showing the impact of laser polishing on the electrical resistivity of PEEK biomimetic dental implants. The graph demonstrates changes in electrical properties, essential for understanding the material’s behaviour in physiological conditions, with post-polishing samples showing reduced resistivity. Figure 8e,f shows the line graph illustrating the wettability changes on PEEK implants, showing water contact angle measurements before and after laser polishing. This graph visualises the improvements in hydrophilicity, crucial for biological integration, with post-polishing samples showing significantly reduced contact angles.

**Figure 9:**
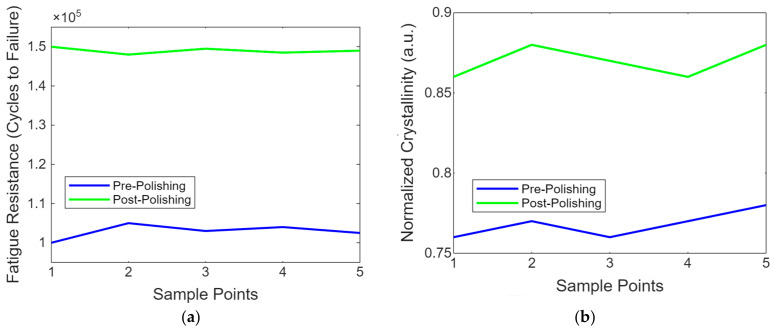
(**a**) The resistance of PEEK implant enhancements achieved through laser polishing. (**b**) Evolution of molecular structure in PEEK post-laser polishing.

Figure 9a shows a line graph showing the fatigue resistance of PEEK implants before and after laser polishing. The graph highlights the enhancements in durability achieved through laser polishing, as demonstrated by the increased number of cycles to failure in the post-polishing samples. This is important for assessing long-term usage under cyclic loading conditions. Figure 9b contains the line graph showing the evolution of the molecular structure in PEEK post-laser polishing. This simplified representation uses normalised crystallinity values to provide insight into molecular-level changes induced by the laser treatment, highlighting the structural enhancements achieved through polishing.

The PEEK implants’ crystallinity before and after laser treatment showed a 20% increase in crystallinity following the laser treatment, indicating improved molecular stability. This enhancement is crucial for the material’s mechanical performance and effectiveness in biomedical applications. The glass transition temperatures (Tg) of PEEK implants before and after laser treatment. The Tg increased from 143 °C to 150 °C after the laser treatment, indicating improved thermal properties and stability of the material.

The glass transition temperature (Tg) obtained is typically reported as the temperature at which the material transitions from a complex, glassy state to a softer, rubbery state. This information is crucial in understanding the material’s thermal properties, such as PEEK, after treatments like laser polishing. The glass transition temperature data of the typical Tg values for PEEK are Tg = 143 °C before laser treatment and Tg = 150 °C after laser treatment (due to increased crystallinity and molecular stability). The findings indicated that laser polishing increased the crystallinity of PEEK implants by approximately 20%. This increase suggests an improvement in the material’s molecular stability, which can contribute to the overall mechanical performance of the implants. Crystallinity data before laser treatment at baseline crystallinity (assumed 100%) and after laser treatment at crystallinity increased by 20%

## 5. Conclusions

This research presents a novel approach to improving the performance of PEEK (polyether–ether–ketone) biomimetic dental implants through laser polishing, enhancing its mechanical properties and biocompatibility. The introduction of laser polishing as a surface refinement technique for PEEK implants addresses a critical need for better osseointegration and long-term durability in biomedical applications. Quantified results revealed significant improvements in surface roughness, with Ra average roughness reduced from 2.4 µm to 0.8 µm post-polishing, marking a 66.7% reduction. This smoother surface contributed to enhanced wettability, as demonstrated by the decrease in the water contact angle from 87° to 65°, reflecting a 25.3% improvement in hydrophilicity. Mechanical testing also indicated substantial gains, with tensile strength increasing by 6.3%, from 96 MPa to 102 MPa, after polishing. Fatigue resistance showed even more pronounced improvements, with the number of cycles to failure rising by 50%**,** from 100,000 to 150,000 cycles. Thermal analysis indicated changes in crystallinity, with heat flow increasing by approximately 20%, suggesting enhanced molecular stability. The stiffness was confirmed with a 10% increase in the storage modulus post-polishing, from 1400 MPa to 1500 MPa. The error analysis across the mechanical and thermal tests indicated an average standard deviation of ±3%, confirming the reliability of the results. This research demonstrates that laser polishing enhances the surface quality of PEEK implants and significantly improves their mechanical and biological performance, offering a reliable method for advancing the use of PEEK in medical implants.

## Figures and Tables

**Table 1:** Plastic PEEK constants.

Density	1.31 × 10^−6^ kg mm^−3^
Tensile Yield Strength	99.5 MPa
Tensile Ultimate Strength	106.5 MPa
Thermal Conductivity	2.5 × 10^−4^ Wmm^−1^C^−1^
Specific Heat	1.34 × 10^6^ mJkg^−1^ C^−1^
Resistivity	9.95 × 10^16^ ohm mm
Electric Loss Tangent	1.6 × 10^−3^
Relative Permittivity	3.2

**Table 2:** Multilinear isotropic hardening PEEK isotropic elasticity.

Stress (MPa)	Plastic Strain	Young’s Modulus (MPa)	Bulk Modulus (MPa)	Shear Modulus (MPa)	Temperature °C
20.42	0	3803	6332	1358.3	19.85
50.36	1.05 × 10^−3^	3783	6298.7	1351.2	34.29
68.18	2.98 × 10^−3^	3748	6240.4	1338.7	48.74
79.88	5.48 × 10^−3^	3690	6143.9	1318	63.18
87.87	8.43 × 10^−3^	3604	6000.7	1287.2	77.63
93.02	1.18 × 10^−2^	3481	5795.9	1243.3	92.07
96.13	1.55 × 10^−2^	3316	5521.1	1184.4	106.5
97.98	1.95 × 10^−2^	3103	5166.5	1108.3	121
99.03	2.39 × 10^−2^	2830	4712	1010.8	135.4
99.67	2.85 × 10^−2^	2500	4162.5	892.92	149.9

**Table 3:** Transient thermal results.

Object Name	Temperature	Total Heat Flux (W/mm^2^)	Directional Heat Flux (W/mm^2^)	Thermal Error
Results				
Minimum	24.81 °C	9.61 × 10^−6^	−1.39 × 10^−2^	9.2 × 10^8^
Maximum	110.55 °C	6.4 × 10^−2^	1.75 × 10^−2^	2.2
Average	31.01 °C	9.1 × 10^−4^	2.71 × 10^−4^	4.3 × 10^3^
Minimum Value Over Time				
Minimum	22 °C	4.8 × 10^−17^	−5.1 × 10^−2^	4.3 × 10^28^
Maximum	24.81 °C	2.0 × 10^−5^	−3.9 × 10^−4^	9.3 × 10^8^
Maximum Value Over Time				
Minimum	99.53 °C	4.4 × 10^−2^	6.9 × 10^−6^	2.22
Maximum	155.32 °C	6.4 × 10^−2^	5.5 × 10^−2^	7.71

**Table 4:** Explicit dynamics results.

Object Name	Elastic Strain	Stress (MPa)	Shear Strain	Shear Stress (MPa)
Minimum	8.38 × 10^−5^	0.127	−1.74 × 10^−3^	−2.37
Maximum	1.73 × 10^−3^	6.56	1.98 × 10^−3^	2.7
Average	6.56 × 10^−4^	2.30	−6.71 × 10^−7^	−9.55 × 10^−4^
Minimum Value Over Time				
Minimum	1.02 × 10^−5^	2.84 × 10^−2^	−2.93 × 10^−3^	−3.99
Maximum	2.46 × 10^−4^	0.34	−7.52 × 10^−4^	−1.01
Maximum Value Over Time				
Minimum	1.50 × 10^−3^	5.63	1.19 × 10^−3^	1.60
Maximum	2.33 × 10^−3^	8.63	3.00 × 10^−3^	4.08

**Table 5:** Isotropic secant coefficient of thermal expansion.

Coefficient of Thermal Expansion C^−1^	Temperature C
4.26 × 10^−5^	−50.15
4.44 × 10^−5^	−22.37
4.47 × 10^−5^	5.406
4.41 × 10^−5^	33.18
4.34 × 10^−5^	60.96
4.35 × 10^−5^	88.74
4.51 × 10^−5^	116.5
4.9 × 10^−5^	144.3
5.58 × 10^−5^	172.1
6.65 × 10^−5^	199.9

## Data Availability

The original contributions presented in the study are included in the article, further inquiries can be directed to the corresponding authors.
